# Brain imaging across scales: an interview with Prof. Mark Schnitzer

**DOI:** 10.1117/1.NPh.12.3.030401

**Published:** 2025-07-30

**Authors:** Ganesh Vasan

**Affiliations:** University of Minnesota, Department of Neuroscience, Twin Cities, Minnesota, United States

## Abstract

Ganesh Vasan (University of Minnesota) interviewed Mark Schnitzer (Stanford University) about his pioneering work in brain imaging across scales.

**Figure:**
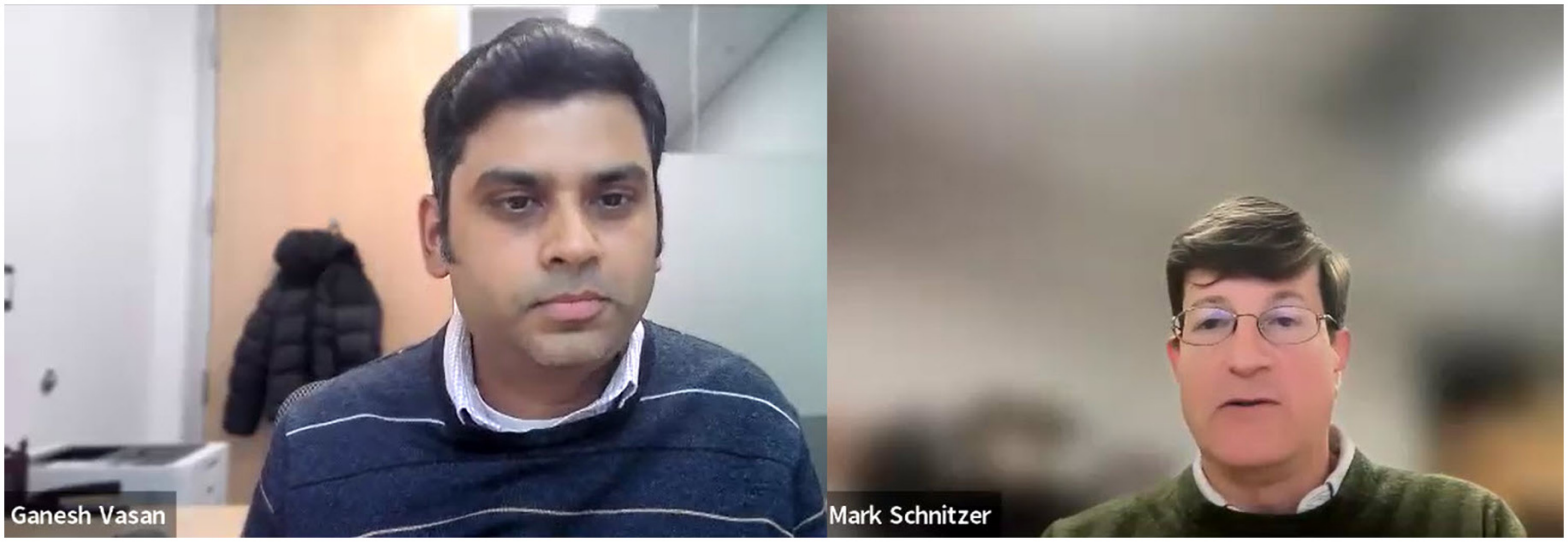
Ganesh Vasan (University of Minnesota) interviews Mark Schnitzer (Stanford University) about his pioneering work in brain imaging across scales. View a video recording of the interview at https://doi.org/10.1117/1.NPh.12.3.030401.

**Figure:**
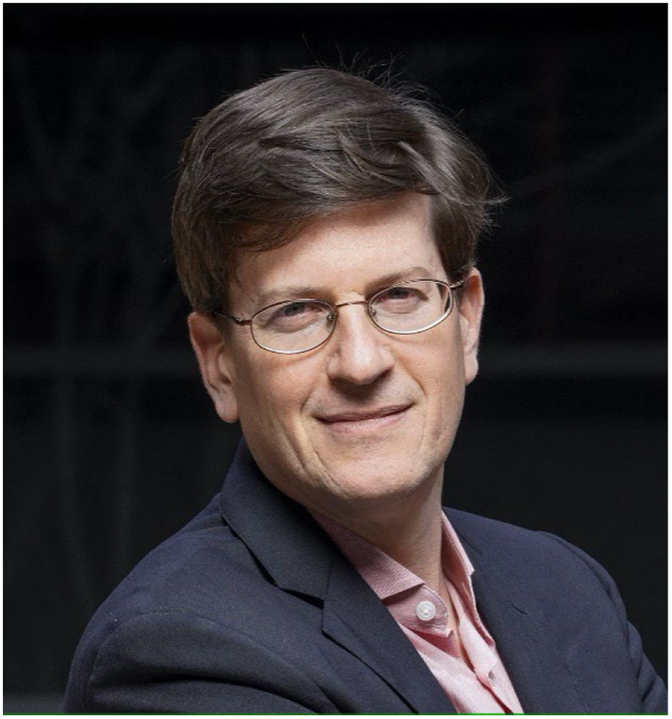
Mark Schnitzer is a professor of biology and applied physics at Stanford University, and an investigator of the Howard Hughes Medical Institute. His research concerns the innovation of novel optical imaging technologies and their use in the pursuit of understanding neural circuits. The Schnitzer lab has invented two forms of fiber-optic imaging, one- and two-photon fluorescence microendoscopy, which enable minimally invasive imaging of cells in deep brain tissues. By combining imaging, electrophysiological, behavioral, and computational approaches, the lab seeks to understand cerebellar dynamics underlying learning, memory, and forgetting. Further work in the lab concerns neural circuitry in other mammalian brain areas such as hippocampus and neocortex, as well as the neural circuitry of Drosophila.

